# Ethogram of Immature Green Turtles: Behavioral Strategies for Somatic Growth in Large Marine Herbivores

**DOI:** 10.1371/journal.pone.0065783

**Published:** 2013-06-19

**Authors:** Junichi Okuyama, Kana Nakajima, Takuji Noda, Satoko Kimura, Hiroko Kamihata, Masato Kobayashi, Nobuaki Arai, Shiro Kagawa, Yuuki Kawabata, Hideaki Yamada

**Affiliations:** 1 Graduate School of Informatics, Kyoto University, Sakyo, Kyoto, Japan; 2 Southwest Fisheries Science Center, National Marine Fisheries Service, National Oceanic and Atmospheric Administration, La Jolla, California, United States of America; 3 Graduate School of Environmental Studies, Nagoya University, Chikusa, Nagoya, Japan; 4 Research Center for Subtropical Fisheries, Seikai National Fisheries Research Institute, Fisheries Research Agency, Ishigaki, Okinawa, Japan; 5 Graduate School of Agriculture, Kyoto University, Sakyo, Kyoto, Japan; 6 Nature and Science Programs, NHK Enterprises, Inc., Shibuya, Tokyo, Japan; 7 Institute for East China Sea Research, Nagasaki University, Nagasaki, Japan; University of Western Australia, Australia

## Abstract

Animals are assumed to obtain/conserve energy effectively to maximise their fitness, which manifests itself in a variety of behavioral strategies. For marine animals, however, these behavioral strategies are generally unknown due to the lack of high-resolution monitoring techniques in marine habitats. As large marine herbivores, immature green turtles do not need to allocate energy to reproduction but are at risk of shark predation, although it is a rare occurrence. They are therefore assumed to select/use feeding and resting sites that maximise their fitness in terms of somatic growth, while avoiding predation. We investigated fine-scale behavioral patterns (feeding, resting and other behaviors), microhabitat use and time spent on each behavior for eight immature green turtles using data loggers including: depth, global positioning system, head acceleration, speed and video sensors. Immature green turtles at Iriomote Island, Japan, spent an average of 4.8 h feeding on seagrass each day, with two peaks, between 5∶00 and 9∶00, and between 17∶00 and 20∶00. This feeding pattern appeared to be restricted by gut capacity, and thus maximised energy acquisition. Meanwhile, most of the remaining time was spent resting at locations close to feeding grounds, which allowed turtles to conserve energy spent travelling and reduced the duration of periods exposed to predation. These behavioral patterns and time allocations allow immature green turtles to effectively obtain/conserve energy for growth, thus maximising their fitness.

## Introduction

Animals are assumed to obtain/conserve energy effectively thus maximising their fitness, which manifests itself in a variety of behavioral strategies [1], [2]. To appropriately assess how animals behave for maximising their fitness, for example, whether they follow optimal foraging theory or not, detailed spatio-temporal behavioral patterns (e.g. feeding, resting, moving) and time allocations to each behavior (i.e. ethogram) should be examined. Making an ethogram by direct observation is fundamental to understanding animal behavior [3]. However, this approach is not feasible for some marine animals that often spend considerable portions of their lives underwater and therefore outside the scope of observational studies [4]. Recently, biologging techniques–monitoring methods that record an animal’s movements, behaviors, physiology and/or environment–using high-resolution devices such as global positioning systems (GPS), acceleration and video loggers have been developed [5]. In brief, while depth loggers have been available for a considerable amount of time (20 years), depth profiles alone do not allow definitive identification of the behaviors being performed. It is the advent of new measurements (e.g. activity [6], jaw movements [7], video [8] etc.) that improve the ability to resolve the function of different dive types. Collectively, these techniques provide an appropriate approach for determining the behavioral strategies of marine animals.

For generalist herbivores, general optimal foraging theory [9], [10] still applies, but is simpler because their food does not move away and thus they do not require capture effort [11], although they do expend considerable energy travelling to patches and selecting high-quality food. Therefore, herbivores can more easily obtain energy resources in foraging grounds. Terrestrial herbivores are known to spend a higher proportion of their time feeding with shorter periods of sleep, to meet their daily energy requirements because of the relatively low nutrient and energy contents of plant-based diets [12], [13]. For marine herbivores, however, only a few studies (e.g. [14], [15]) based on detailed spatio-temporal behavioral patterns have investigated how marine herbivores behave (e.g. feeding, resting and moving) and allocate the time to each behavior to obtain/conserve energy effectively. The deficiency in spatio-temporal research is due to the lack of high-resolution monitoring techniques for studying animal behavior in marine environments, although a number of studies have focused on the behavior of such subjects (e.g., sea urchin, fish, sea cow, marine iguana and green turtle (*Chelonia mydas*) [14]–[19]).

Here, we report the fine-scale behavioral patterns, microhabitat use and time allocations for individual behaviors in immature green turtles using the combination of novel high-resolution biologging techniques. Green sea turtles are the only reptiles that consume seagrass as well as marine algae [20]. Previous biotelemetry and biologging studies have revealed that they have relatively small home ranges, migrate between foraging and resting sites and use reef ledges and benthic habitats as resting sites [8], [21]–[25]. To date most of the focus for biotelemetry and biologging studies has been on adult green turtles. For example, in some breeding areas where food is scarce adults spend long periods resting on the seabed, typically at >10 m, while in breeding areas where food is available they forage in very shallow (<2 m) water [26]. These studies show how behavior in this species is plastic and can be modified depending on local resources. However little is known about how immature green turtles behave. During the immature stage, green turtles do not need to allocate energy to reproduction and are at low rate of predation from sharks [27]. Therefore, green turtles at the immature stage are assumed to select/use feeding and resting sites mainly to maximise their fitness in terms of somatic growth and the maintenance of body condition, although turtle behavior might be influenced by predation risk [28]. In the present study, we identified feeding, resting and other behaviors of immature green turtles from dive depth, head acceleration and video data collections, we identified their diel behavioral patterns and time allocations, and we determined how these patterns relate to their somatic growth strategy.

## Materials and Methods

### Study Area and Experimental Animals

This study was conducted in the southwestern area of Iriomote Island (24°17’ N, 123°53’ E), which is one of the Yaeyama Islands in southwestern Japan. Most areas of the Yaeyama Islands have been designated a national park. The Yaeyama Islands include the largest coral reef ecosystem in Japan [29], although seagrasses and patches of marine algae are also present in sandy regions of the inner reefs [30]. The predominant seagrass species include *Thalassia hemprichii*, *Cymodocea serrulata*, *C*. *rotundata*, *Syringodium isoetifolium*, *Enhalus acoroides* and *Halophila ovalis* [30], while marine algae include *Dictyota* sp., *Hypnea pannosa* and *Digenea simplex* [31]. Immature and mature green turtles are known to inhabit these coastal areas [32], [33].

We studied eight immature green turtles that were hand-captured by a local fisherman with permission from the Fisheries Adjustment Commission of Okinawa Prefecture (Permits 21–2 and 23–2) (Table 1). Before the experiments, the turtles were placed in a 500 L tank for 2 to 17 days at the Research Center for Subtropical Fisheries, Seikai National Fisheries Research Institute at the Fisheries Research Agency, Japan, where we took physical measurements, determined the conditions for feeding events from head acceleration data (see “Definition of feeding bouts based on depth data”) and assessed any negative impacts that might have been caused by the equipment.

**Table 1:** A summary of physical, experimental and behavioral performance data for green turtles used in this study.

Turtle	SCL	BW	Sensor	Period of depth	Period of HA	No. of	No. (duration)	Duration of	Max. speed
ID	(cm)	(kg)	deployed	data used (h)	/V data used (h)	dives	of feeding dive	feeding bout (h)	(km/h)
							extracted from HA data	extracted from D data	
CM 1	49.2	24.3	D, T, S, HA	0.0	0.0	0.0	0	0	3.02
CM 2	60.9	29.2	D, T, S, HA	117.0	67.0	759	147 (6.5 h)	23.8	4.54
CM 3	72.5	50.2	D, T, G, V	40.0	4.0	327	**−**	2.9	**−**
CM 4	59.6	26.8	D, T, G, V	24.0	5.0	430	**−**	9.0	**−**
CM 5	54.3	24.1	D, T, G, V	87.0	10.5	406	**−**	11.7	**−**
CM 6	61.5	26.3	D, T, G, V	96.0	0.7	439	**−**	15.5	**−**
CM 7	59.8	29.1	D, T, G, V	109.0	0.0	520	**−**	21.5	**−**
CM 8	60.2	29.4	V	**−**	4.9	**−**	**−**	**−**	**−**

Values in parentheses represent the period of head acceleration or video data used. For the recording period of video data, see Table S1. SCL, straight carapace length; BW, body weight; D, depth; T, temperature; S, speed; HA, head acceleration; G, GPS; V, video.

### Instruments and Experimental Protocol

The experimental protocol was approved by Animal Research Committee of Kyoto University (No. 22–4). We used various types of data loggers (Table 1). To monitor the feeding events of turtles CM1 and CM2, we attached small multi-sensor data loggers (M190L-D2GT: 15 mm diameter, 53 mm length, 17 g, Little Leonardo Co., Tokyo, Japan) that recorded depth, temperature and two-axis (surge and heave axes) acceleration to the tops of the turtles’ heads. This approach was modified from the methods of Okuyama et al. [34], where they attached an accelerometer to the beak (rhamphothecae), to allow us to retrieve the data loggers more easily. Before this experiment, we confirmed the validity of this modified method by conducting tank experiments that followed the same procedure as Okuyama et al. [34]. To monitor dive behavior for CM1 and CM2, multi-sensor data loggers (W1000–3MPD3GT: 26 mm in diameter, 175 mm in length, 140 g in air, 48 g in water, Little Leonardo Co.) were attached to the turtles’ carapaces that incorporated a water pressure sensor, a swim speed sensor and a temperature sensor. The sampling interval was 1 s for all parameters except for the head acceleration sensors, which recorded at intervals of 1/32 s.

For the remaining turtles, CM3 to CM8, we directly observed feeding events by attaching video loggers (GoPro HD®, Woodman Labs, CA, USA) in custom-made waterproof cases with recording scheduled system and extra-battery (Logical Product Co., Fukuoka, Japan) to their carapaces (Table 1), which was 72×56×88 mm^3^ in dimension and 360 g in air, 55 g in water. Video was recorded at 30 frames per second and was multi-scheduled to record during various time periods within the range from 5∶30–18∶00, except for CM5, which recorded from 19∶00–5∶30 (See Table S1). Moreover, to monitor the vertical and horizontal movements of the turtles, CM3 to CM8, Fast-loc GPS and depth tags (Mk10-F, Wildlife Computers, WA, USA: 86×83×32 mm^3^, 180 g in air (50 g in water)) were attached to their carapaces (Table 1). Depth and temperature data were recorded at 10 s intervals, and GPS location was fixed almost every time a turtle surfaced to breathe. Locations from Fast-loc GPS loggers are generally accurate to within a few tens of meters [5]. Only a video logger was attached to CM8 because we had a limited number of instruments (Table 1).

To retrieve data loggers from released turtles, data loggers were equipped with floats made of copolymer foam (Nichiyu Giken Kogyo, Saitama, Japan) in which a VHF transmitter (130 BB, Advanced Telemetry Systems, Isanti, MN, USA) and a time-scheduled release mechanism (Little Leonardo Co.) were embedded. These logger units were affixed to the centre of the carapace or the head of a turtle using epoxy putty (Konishi Co., Ltd. Osaka, Japan), two-component epoxy resin (ITW Industry Co., Ltd. Osaka, Japan) and a plastic cable tie that was connected to the time-scheduled release mechanism. The logger units were designed to weigh <4% of a turtle’s weight. Although the logger units increased drag (as occurs with Argos transmitters [35], [36]), no behavioral changes were observed and normal feeding was noted during visual observations during preliminary attachment tests in the holding tank.

Turtles equipped with logger units were released very close to their initial capture point. The time-scheduled release mechanisms were programmed to activate 8–168 h after release, at which time an electric charge would incise the plastic cable. Then the logger units would detach from the turtles and float to the sea surface. All of the logger units were located by radio telemetry using radio receivers and a four-element Yagi antenna (FT-290mk-II/AR; Yaesu Musen Co. Ltd., Tokyo, Japan).

### General Data Analysis

The time-series data obtained were analysed using the software IGOR Pro ver. 6.2 (WaveMatrics, Inc. Lake Oswego, OR, USA) and Ethographer ver. 2.00 (K.Q. Sakamoto, Hokkaido University, Sapporo, Japan, for details, see [4]). A dive was defined as an event that exceeded a depth of 0.5 m for at least 30 s, and dive parameters were extracted for each dive. Dive depth was defined as the maximum depth during a dive. To eliminate erratic behaviors caused by capture-related stress, we only used data that were collected after a first consecutive U-dive at a similar depth after the release, which seemed to indicate reversion to natural behavior in released green turtles (Table 1). Thus, CM1 was eliminated from our analysis because it did not demonstrate consecutive U-dives at the same depth, perhaps due to short monitoring period.

Acceleration sensors provide information on both dynamic (e.g., flipper movement and head motion) and static acceleration (e.g., posture of the body part to which the data logger is attached) which are represented as high and low frequency acceleration signals, respectively [37]. Thus, to separate head motion and head angle, dynamic and static accelerations in the surging and heaving axes were separated using a 0.32 Hz low-path filter (IFDL ver 4; WaveMatrics), using the value defined from the observation of feeding behavior in the tank prior to the experiments.

### Definition of Feeding Bouts Based on Depth Data

The extraction of feeding behavior from acceleration data followed the same procedure of Okuyama et al. [34]. For turtles CM1 and CM2, the feeding signal was defined as an acceleration >+1.5 or <−1.5 m s^−2^ in surging acceleration and >+1.0 or <−1.0 m s^−2^ in heaving acceleration in the head motion. When two feeding signals occurred continuously within 5 s of each other, they were considered one ‘feeding event’. To eliminate noise pulses caused by contact with obstacles and breathing behaviors, we removed from the analysis feeding signals that did not continue for >1 s or occurred at <0.2 m of depth. Under these conditions, we determined that the detection rate of feeding events from head acceleration data was nearly perfect (96%: 26 of 27) and that the false detection rate was 16% (4 of 25) by comparing the acceleration data to the visual observation of feeding events (Okuyama et al. unpublished data). Detailed descriptions of the extraction methods of feeding events from head acceleration data are given in Okuyama et al. [34].

**Figure 1:**
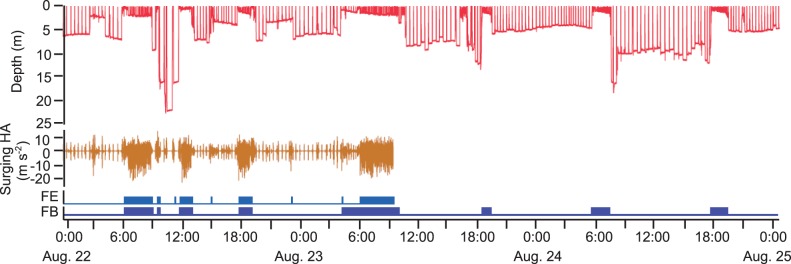
The extraction results of feeding behaviour from head acceleration data. (A) Depth distribution of feeding events by immature green turtles calculated from head acceleration data. (B) Distribution of the total time period of feeding events at each depth. (C) Depth distribution of feeding dives (dives with feeding events). (D) Distribution of the duration of consecutive feeding dives occurring in shallow water than 3 m.

If head acceleration data was not recoreded at the same time as depth data, it was still possible to identify feeding behaviors solely from depth data. Initially, we had acceleration data and depth data, we defined ‘feeding dives’, which were dives that contained ‘feeding events’ determined from head acceleration data. Secondly, when we had both sets of data, using the analysis of the characteristics of a ‘feeding dive’ from the head acceleration data (see Results, and Fig. 1), a ‘feeding bout’ in a dive depth profile was extracted as follows: (1) a feeding bout consisted of a series of U-shaped dives with a fluctuation of depth profile in the flat bottom phase (Type 1b in the definition of dive types by Houghton et al. [38]), (2) the depth was less <3 m during >80% of the dive duration, and (3) the consecutive dives continued for more than 30 min. This ‘feeding bout’ was defined as feeding behavior extracted from depth data. For accuracy evaluation of a feeding bout, see Results and Fig. 1. Therefore, we were able to apply this same extraction criteria for dive depth data when we did not have head acceleration data.

Moreover, to understand the behavioral patterns of the turtles, their behaviors were classified into three phases: feeding, resting and other. A feeding phase was a period with feeding bouts and a resting phase was a period during which a turtle performed U-shaped dives, excluding feeding phases. Other phases were periods during which turtles did not perform U-shaped dive, but other kinds of behaviors (Type 2–5 in the definition of dive types by Houghton et al. [38]), including swimming.

### Location Data Analysis

To remove erroneous locations, GPS tracks were filtered using a maximum swimming rate of 5 km h^−1^, which was defined by the data obtained from the speed sensor, between successive locations (see Results, Table 1). In addition, data were removed when a location occurred on land. We estimated individual home ranges using 95% fixed kernel (FK) and 50% FK isopleths [39] using ArcGIS 10 (Environmental Systems Research Institute, Inc., Redlands, CA). We used least squares cross-validation to select the kernel smoothing factor [40]. We also estimated the utilisation area for each feeding, resting and other phase, with 100% minimum convex polygon (MCP) ranges using ArcGIS 10 because the amount of location data in each phase was not sufficient for calculating the FK estimation.

To determine the distance between resting and feeding sites, the distance was calculated between the GPS location where a resting phase terminated and the point where the next feeding phase started, and vice versa. Moreover, a straightness index (SI) was calculated during periods when turtles were coming to and going from seagrass meadows, as well as during feeding and resting phases. SI was calculated as the ratio between the straight-line distance from the start to the end of a turtle’s route and the total length of the route [41]. In cases where the phase changed directly from resting to feeding, and vice versa, SI was not calculated because the SI value was 1.

Since feeding bouts occurred in the shallower waters (see Results), to determine the effect of tidal level on the approaches of turtles to inner reef sites, tidal level data every hour for Iriomote Island were downloaded from the website of the Japan Meteorological Agency (http://www.data.kishou.go.jp/db/tide/suisan/index.php). Then, we investigated the relationships between the occurrence of feeding behavior and time, tidal levels, or tidal movement using generalized linear model (GLM) with logistic link-function. The ocurrence of feeding behavior was treated as an explanatory variable, while time, tidal levels and tidal movement were dependent variables. These variables were extracted in each hour, and in total 473 h of data were used (Table 1). Since the ocurrence of feeding behavior (occurrence or not), time (0–23 h of a day) and tidal movement (rising tide, falling tide and no tidal movement) were categorical data, the dummy variable was used to do GLM analysis. We used the ‘glm’ package and Wald-test in the software R 2.10.1 [42] to assess the effect of these variables on the occurrence of feeding behavior. In addition, to determine the effect of body condition on the duration of feeding bouts, a body condition index (body mass/[straight carapace length]^3^) was calculated [43].

## Results

The physical, experimental and behavioral data obtained are summarised in Table 1. A total of 473 h of dive data, 67 h of head and body acceleration data, 356 h of GPS locations and 25.1 h of video data were obtained. Because the video logger attached to CM7 spontaneously detached before recovery, we did not obtain video data from CM7. The maximum swimming rates of CM1 and CM2 were 3.02 and 4.45 km h^−1^, respectively.

### Feeding Behavior Extracted from Head Acceleration

**Figure 2:**
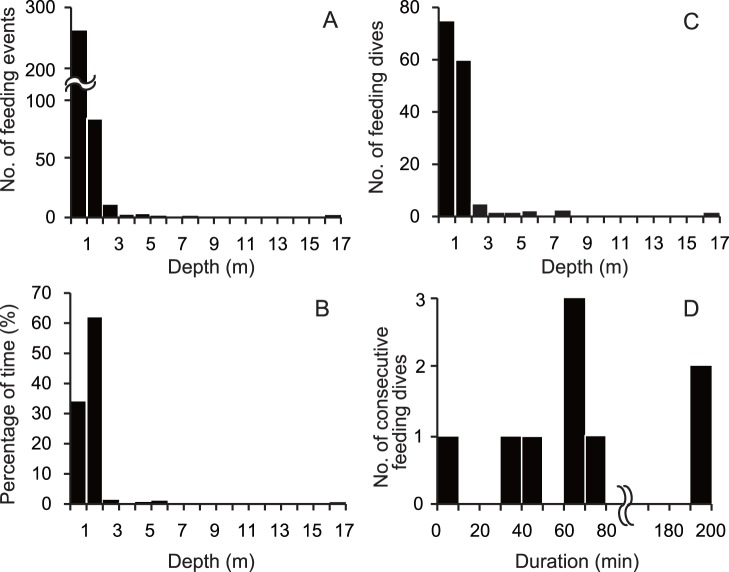
A typical profile of dive depth and surging head acceleration (HA) for an immature green turtle (CM2). The horizontal bars at the bottom represent the timing of feeding events (FE) and feeding bouts (FB), respectively. The recording of head acceleration terminated at 9∶23 on August 23th.

A total of 376 feeding events were extracted from the head acceleration data for CM2 during 67 h of deployment, while no feeding events by CM1 were observed, likely due to the short duration of the recording period. The feeding events were classified into two categories: those that occurred at depths shallower than 3 m and those that occurred at depths deeper than 3 m (Figs. 1 and 2). Most feeding events (96.0%; 361 of 376) occurred at depths shallower than 3 m (Fig. 1A), which made up 97.5% of the time spent feeding (Fig. 1B). These shallow-water feeding events were characterised by consecutive occurrences in a dive, while feeding events occurred sporadically at depths deeper than 3 m (Fig. 2).

Most feeding dives extracted from the head acceleration data (94.5%; 139 of 147) occurred at depths shallower than 3 m (Fig. 1C) and were characterised by a U-shaped profile with fluctuations of depth profile in the flat bottom phase of the dive (Type 1b in the definition of dive types by Houghton et al. [38]). Most feeding dives in water shallower than 3 m followed each other without interruptions (Fig. 2). Among these consecutive feeding dives (*N* = 9), the duration was 84.7±69.6 min, and 89% (eight of nine) were longer than 30 min (Fig. 1D). The remaining one was not continuous with other feeding dives, but rather with consecutive shallower dives without feeding events that lasted until the next feeding dive (Fig. 2). Among the shallow-water feeding dives, 87.4±15.5% of the time period was spent during feeding events in a single dive. Among the remaining eight feeding dives that occurred at depths deeper than 3 m, three feeding dives including four feeding events were also characterized by a U-shape profile with fluctuations in the flat bottom phase, where 8.2±4.2% of the time period was spent for feeding events. Meanwhile, the remaining five feeding dives including 11 feeding events were characterized by complete U-shaped profile without fluctuation, where 4.1±3.5% of the time period was spent for feeding events.

### Feeding Behavior Extracted from Video Data

**Figure 3:**
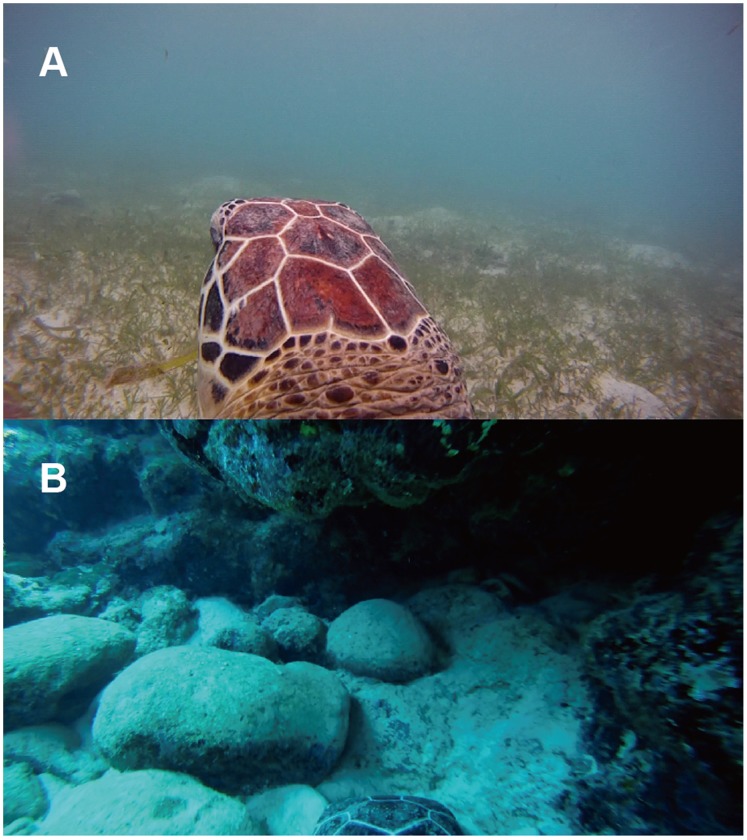
Video images of typical behaviour by the immature green turtles. (A) Feeding behaviour of CM8 on seagrass in shallow water and (B) resting behaviour of CM4 under the reef ledge. Video clips from which the images were taken are available in the Movie S1 and S2, which is available online.

Out of 25.1 h of video data, CM8 migrated from the reef ledge to the seagrass meadow in shallow water, and then started feeding at 9∶07 (Figs. 3A and Movie S1). Feeding continued until at least 12∶01, but the termination time could not be confirmed because the video recording stopped due to low battery power. The food items could not be identified, but they likely included some species of seagrass and *Halophila* sp. (*ovalis*), based on their shapes. In the seagrass meadow, the turtle spent most of its time feeding in the seagrass, except when rising to the surface to breathe. During feeding, the turtle continuously moved its head to bite and then chew the food, which corresponded to the head movement pattern that was speculated from the acceleration data (Fig. 2) and feeding observation in the tank. Also, we observed three sporadic feeding events by CM3 on a jellyfish (*Scyphozoa*) at 8∶18–8∶22 during swimming at a depth of 5.5–7.5 m. Moreover, CM5 bit and then chewed something that was on a hard coral at 19∶17 while resting at a depth of 17.2 m; this food item could not be identified because it was outside the image frame. No feeding events were observed in the video data of the other turtles.

### Evaluation of Feeding Bouts

We extracted 8 feeding bouts, which consisted of 172 dives, during the period when both depth and head acceleration were recorded. These feeding bouts included all of the 139 feeding dives that occurred at depths shallower than 3 m, which were extracted from head acceleration data, while the remaining 33 non-feeding dives occurred before feeding began, after it concluded or between feeding events (Fig. 2). Each of the feeding bouts always included any feeding dives determined from head acceleration data.

### Behavioral Pattern and Time Allocation for Each Behavior

Following release, all of the turtles remained at the same location after completing their first subsequent U-shaped dives. The utilisation distributions were 141.7±121.3 ha and 59.1±50.5 ha for the 95% and 50% FK isopleths, respectively (Table 2).

**Table 2:** A summary of home range area estimates and horizontal movement performance during the entire period and during the three phases (feeding, resting and other) for green turtles.

	CM 3	CM 4	CM 5	CM 6	CM 7
**Whole phase**					
N	223	193	281	423	500
95%/50% FK (ha)	108.3/52.7	53.5/30.9	119.1/31.5	73.9/32.5	353.5/148.0
**Feeding phase**					
N	30	128	125	149	242
# bouts	1	1	7	9	9
# place utilized	1	1	1	1	1
95%/50% FK (ha)	45.2/28.9	39.8/19.3	101.6/29.9	47.7/24.0	267.2/108.3
MCP of a bout (ha)	5.5	6.3	3.8±2.7	1.1±0.6	47.3±49.2
Straightness Index	0.06	0.02	0.30±0.25	0.21±0.21	0.32±0.32
**Resting phase**					
N	97	43	102	145	153
# bouts	6	4	7	11	11
# place utilized	3	2	1	2	5
95%/50% FK (ha)	59.6/28.6	36.9/20.0	34.5/12.6	49.3/21.7	136.5/48.1
MCP of a bout (ha)	0.5±0.6	0.3±0.1	0.7±0.6	0.4±0.4	0.7±0.8
Straightness Index	0.30±0.21	0.19±0.13	0.09±0.07	0.11±0.08	0.32±0.32
**Other phase**					
N	96	22	54	129	105
# bouts	7	6	13	20	19
95%/50% FK (ha)	75.3/51.8	37.8/22.9	110.9/49.6	76.0/42.3	263.1/160.0
**From resting to feeding places**					
Distance (km)	0.09	0.12	0.37±0.26	0.11±0.11	0.37±0.33
Straightness Index	–	0.86	0.73±0.21	0.58±0.43	0.72±0.20
**From feeding to resting places**					
Distance (km)	0.11	0.14	0.33±0.21	0.21±0.27	0.30±0.21
Straightness Index	–	0.73	0.68±0.30	0.65±0.02	0.66±0.29

Values are presented as mean ± standard deviation. FK, fixed kernel density estimates, MCP; minimum convex polygon estimates.

**Figure 4:**
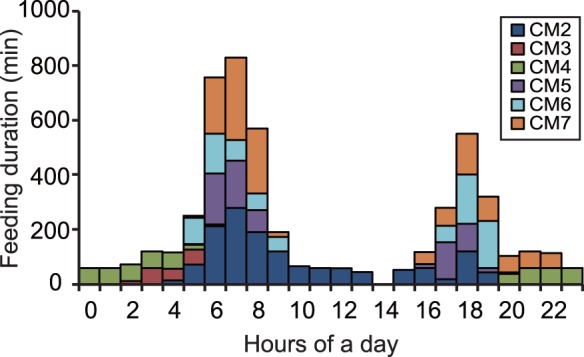
Diel pattern in the time spent during feeding bouts by each of the immature green turtles.

**Figure 5:**
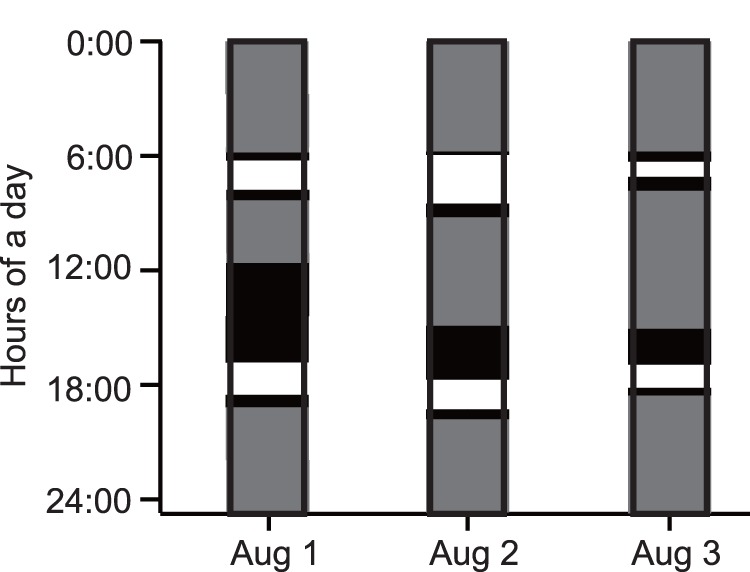
Daily ethogram of an immature green turtle (CM5) over 3 days. The ethogram consists of three phases, feeding (white), resting (grey) and other (black), which were defined from the depth data (see Materials and Methods).

**Figure 6:**
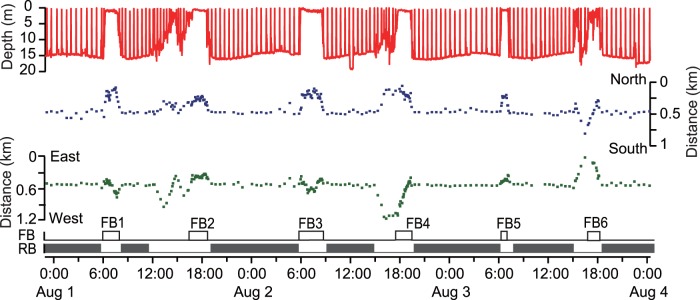
Typical profiles of vertical (depth) and horizontal (north-south and east-west distance) movements by an immature green turtle (CM5). Horizontal bars at the bottom indicate the period of feeding resting and other phases, respectively.

**Figure 7:**
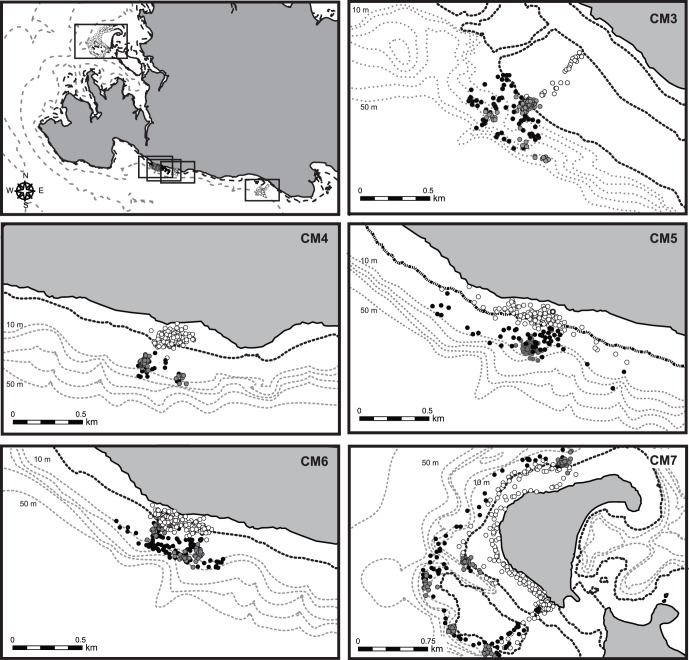
A map of the study sites at Iriomote Island, and GPS locations for five immature green turtles during three phases. White, grey and black circles represent feeding, resting and other phases, respectively. Solid and dotted lines represent the low-tide and 10 m interval depth contour lines, respectively.

**Figure 8:**
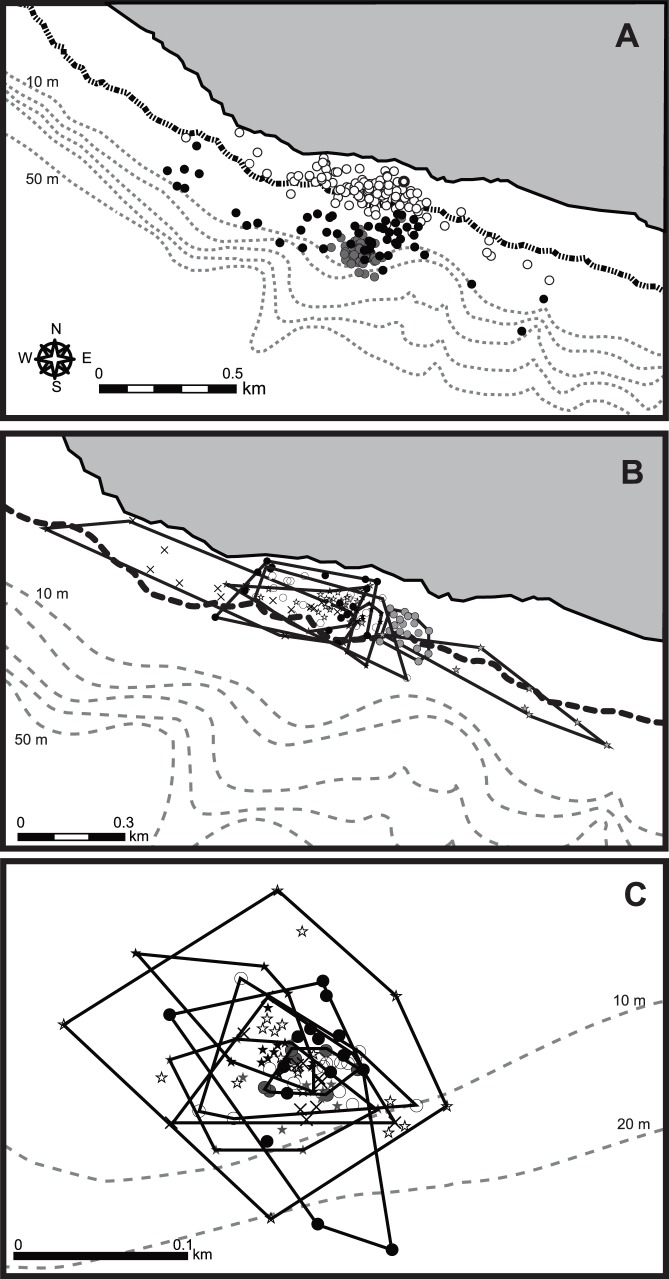
GPS locations for an immature green turtle (CM5) during three phases. (A) feeding (white), resting (grey) and other phases (black). Black and grey dotted lines represent the low-tide and 10 m interval depth contour lines, respectively. GPS locations during (B) feeding and (C) resting phases. Symbols and solid lines represent each phase and its area within a 100% minimum convex polygon.

In total, 41 feeding bouts that took 84.4 h were extracted from depth data in six turtles (CM2–7). In individual turtles, feeding bouts occupied 19.1±10.1% of their time. The duration of a feeding bout was 2.1±1.5 h. The occurrence pattern of feeding bouts showed two peaks within a day, with concentrations between 5∶00 and 9∶00 and between 17∶00 and 20∶00; individuals mostly rested between bouts (Figs. 4 and 5, Table S2). Only one feeding bout occurred at night under moonlight, by CM4 (Fig. 4). GPS data showed that the turtles started to move into shallower areas at the beginning of each feeding bout, and that they generally stayed in intertidal areas during these events (Figs. 6 and 7). The area of activity for all of the feeding bouts largely overlapped in each turtle, but during each bout, the turtles used slightly different locations (Fig. 8B and Table 2). The total area used during feeding bouts by an individual turtle was 141.7±121.3 ha and 59.1±50.5 ha for the 95% and 50% FK isopleths, respectively (Table 2). The result of GLM analysis showed that the occurrence of feedinng behavior was significantly affected by time especially 5–9 h and 17–20 h of a day, and not by tidal level and tidal movement (Table S2). Also, there was no significant relationship between the duration of feeding and tidal level (*ANOVA*, *N* = 41, *F*_1,40_ = 1.00, *P* = 0.32). Moreover, mean duration of feeding in individual turtles was not significantly related with the body condition index (*ANOVA*, *N* = 6, *F*_1,5_ = 0.23, *P* = 0.66).

Outside of feeding phase, the turtles generally engaged in resting behaviors, which were represented by U-shaped dives and made up 68.7±10.9% of their time (Fig. 5, 6). The turtles mostly rested at night, but also around midday, between the two peaks in feeding (Fig. 5, 6). There was no doubt that U-shaped dives were resting behaviors from the video data, which showed that the turtles either did not move or remained still and looked around while on or under the reef ledge (Figs. 3B and Movie S2). However these periods included some feeding (3.4% or 5 of 147 feeding dives from head acceleration data, and 0.6% or 1 min within the 2.9 h feeding periods from the video data). Each turtle displayed resting phase 6–11 times during the experiment; the locations used during these phases were concentrated within 1–5 places, indicating that turtles used the same places for resting, although it was not always the same spot (Figs. 7 and 8C, and Table 2). The area used across all resting bouts by individual turtles was 63.4±42.1 ha and 26.2±13.5 ha for the 95% and 50% FK isopleths, respectively (Table 2).

During the ‘other’ phase, which made up only 12.2±7.0% of their time, turtles moved vertically and horizontally (Fig. 5, 6). Of this phase, 73.8% (48 of 65) occurred before or after feeding bouts and represented travel to shallow areas, but occasionally it included wandering movements before or after this behavior (Fig. 7). Mean distance and the SI from a resting place to the next feeding ground were 0.27±0.25 km and 0.71±0.24, and from a feeding ground to the next resting place they were 0.27±0.30 km and 0.67±0.25, respectively. On the other hand, 26.2% of this phase (17 of 65) was not related to feeding bouts; it occurred either between periods in the same resting place or between periods in two different resting places. The SI value during the other phase, which was not related to foraging movements, was 0.38±0.34, whereas during feeding and resting phases the values were 0.32±0.24 and 0.21±0.22, respectively (Table 2). The SI values differed significantly among periods of transit to seagrass meadows, during non-transit movements and during feeding and resting phases (One-way ANOVA, *F*_4,104_ = 16.7, *P*<0.0001). Three sporadic feeding events by CM3 on a jellyfish that were observed by the video logger, and four feeding events by CM2 that were observed by the head acceleration were included in this phase. The area used during all of the other phases in individual turtles was 112.6±88.0 ha and 65.3±54.2 ha for the 95% and 50% FK isopleths, respectively (Table 2).

## Discussion

### A Feeding Bout as an Indicator of Feeding Behavior

This study quantitatively demonstrated the behavioral patterns, microhabitat use and time allocation of green turtles using high-resolution tracking with acceleration and GPS loggers, and direct observation with a video logger in the wild. Head acceleration and video data demonstrated that the turtles at Iriomote Island were mostly herbivorous and that their feeding was focused on seagrass, which corroborates data from the anatomical research for the stranded turtles in the Yaeyama Islands [44]. An evaluation of feeding bouts and horizontal movements during feeding bouts indicated that feeding bouts were definitely foraging trips to seagrass meadows in intertidal areas. In some regions, it has been reported that green turtles are largely dependent on invertebrates (e.g. [45], [46]) In this study, our video logger confirmed the consumption of jellyfish and an unidentified food item on a hard coral. However, invertebrate feeding events occupied only 5 min (2.8%) within 2.9 h of feeding. Moreover, although we monitored 15 sporadic feeding events that occurred in water deeper than 3 m from the analysis of head acceleration, these events constituted a small fraction of the total events (4.0%) or time (2.5%).

### Timing and Duration of Feeding

The turtles spent an average of 4.8 h feeding each day, with each bout lasting 2 h on average; this was a much longer feeding duration per bout than has been reported elsewhere [8], [47]. This difference may be due to differences in primary food items between this study (seagrass) and previous studies (algae, jellyfish and macroplankton). The utilisation area of seagrass meadows generally overlapped; however, the area used during each feeding bout was slightly different, indicating that there was a strategy for conserving food resources or a lack of extreme site preferences among feeding grounds. The occurrence pattern for feeding bouts contained two peaks, between 5∶00 and 9∶00, and between 17∶00 and 20∶00, showing that the feeding of immature green turtles is crepuscular. To date, some studies have described foraging patterns of green turtles (Table 3), although the monitoring methods of these previous studies did not have sufficient spatio-temporal precision like this study. Green turtles at Mayotte Island [24] and in San Diego Bay [48] foraged in seagrass meadows during the day. Meanwhile, the foraging pattern of turtles at Great Inagua [19], St. Croix, US Virgin Islands and Mosquito Lagoon, USA [49], included two feeding events during the day, separated by a resting phase at noon, which was very similar to the turtles in our study. Williams [50] reported both patterns at St. John, US Virgin Islands. What factors are responsible for the differences in feeding periods among these studies? Perhaps this difference is because larger turtles are able to continuously feed on seagrass over longer intervals due to their larger gut capacity and faster processing rate [19], [51], while smaller turtles need to wait for digestion around midday. Indeed, in the previous studies, larger turtles tended to feed continuously during the day (Table 3). This suggests that the immature turtles spent as much time feeding as possible to maximise their energy acquisition. This explanation is supported by the fact that the amount of food consumed and digestibility increases with green turtle size, although digestibility is affected by body temperature [19]. In comparison, dugongs (*Dugong dugon*), which are larger than green turtles, appear to spend at least about 16 h feeding each day [52]; and that in large terrestrial herbivores [51]. On the other hand, our conclusion is also supported in avian literature, for example, red knots (*Calidris canutus*) maximise their net rate of intake over time (foraging time plus digestive breaks) while foraging [53]. Moreover, all of the daytime feeding on marine algae in small green turtles may be due to differences in digestion efficiency because complex carbohydrates are very different between algae and seagrass [54]. Another explanation for crepuscular feeding may be the result of predator avoidance in which the turtles move into deeper water to reduce predator encounter rate during the middle of the day when the visibility to predators is high. In the nightime, because visual cues have an important role in sea turtle prey detection [55], they may not feed. However, similar to two other studies [19], [24], one feeding bout was also observed on a moonlit night in the present study. Therefore, although feeding usually occurs between dawn and dusk, it sometimes occurs on nights when the moon is bright enough to allow the turtles to see their prey. Balaz et al. [56] and Seminoff et al. [22] also reported nocturnal use of nearshore habitats to avoid human activities such as heavy boat traffic and fishing pressure, as well as for foraging. Our study site, Iriomote Island, is inside a national park and development is regulated; therefore, there is no human development or roads in the area. Moreover, it is far away from popular recreation areas, so there is little boat traffic and low fishing pressure, although some fishing boat traffic occurs offshore. Therefore, it is unlikely the turtle foraged during the night to avoid human interactions as the turtles in our study area do not seem to be subjected to substantial anthropogenic influences. Because the tidal level and tidal movement did not affect feeding behavior of the turtles during this study, restricted access to seagrass meadows at low tide did not appear to be a limiting factor, as has been reported for other green turtles [24] and dugongs [15]. Body condition index values for our turtles were not related to the mean duration of feeding bouts and, therefore, does not have an influence on feeding duration.

**Table 3:** Comparison of studies on foraging behaviors in green turtles.

n	CL (cm)	BW (kg)	Place	Description of Feeding pattern	Dominant prey species	Reference
12	**–**	8–66	Great Inagua,	Two peaks: 08∶00–10∶00 and 14∶00–17∶00	Seagrass	[19]
			Bahama			
3	36–40*	7.6±1.0	St. Croix,	Two events in a day; In the morning and afternoon	Seagrass	[21]
			US Virgin Islands			
14	**–**	7.8–54.5	Mosquito Lagoon,	Two peaks: in midmorning and midafternoon	Seagrass	[49]
			USA			
5	**–**	4–60	St. John,	Turtles started feeding at 07∶00 to 07∶30, and	Seagrass	[50]
			US Virgin Islands	continue for 9 h, but occasionally sleep at midday		
6	70–102	**–**	Mayotte Island,	Turtles fed during 06∶00–18∶00, and occasionally	Seagrass	[24]
	in CCL		France	fed during the nighttime		
4	34–45	8.3–12.1	Palm Beach,	Turtles visited foraging ground during a daytime	Algae	[23]
	in SCL		USA	(06∶00–20∶00)		
20	55–103	18–147	San Diego Bay,	Turtle visited eelgrass meadows during daytime	Seagrass	[48]
			USA	hours (05∶00–18∶00)		
6	54–73	24–50	Iriomote Island,	Two peaks: 05∶00–09∶00 and 17∶00–19∶00	Seagrass	This study
	in SCL		Japan			

SCL, straight carapace length; CCL, curved carapace length; BW, body weight.

Active feeding in the middle of the water column and stationary feeding have been reported as the main feeding behaviors at other sites [8], [47]. In this study, these types of feeding behaviors were rare, although the turtles spent enough time in benthic habitats and in the middle of the water column for additional feeding to have occurred. Therefore, these feeding behaviors may be not intentional, but may represent opportunistic responses to food availability.

### Microhabitat Use and Behavioral Strategy for Growth

Green turtles are known to reside within stable home ranges near their foraging grounds [49], although on occasion they move quite extensively, presumably in the search for new foraging sites [57]. Similarly, the immature turtles in the present study were most often found along the shallow nearshore margins of the study area and showed habitat affinities with small home ranges. The home ranges in the present study were smaller than those reported in other studies, possibly because of differences in the tracking period, individual size and marine habitats, although calculation of their home range is heavily dependent on the accuracy and the frequency of locations [58], [59]. Short-term home ranges seem to shift in terms of optimal patch use [60]. In fact, Seminoff et al. [22] reported that green turtles used one to three activity centres during 34 to 96 days of tracking, which may result from optimal patch use.

Within such small home ranges, the immature green turtles in the present study spent most of their time resting, followed by foraging trips to seagrass meadows. This behavioral pattern represents a strategy for effectively converting the energy gained from food into growth. Intensive movements to resting places, including return trips between two peaks in feeding intensity, may indicate that turtle feeding grounds have a high risk of exposure to predators (see above)[28]. The turtles selected resting places that were close to their feeding grounds, allowing them to save energy for travelling and reducing the duration of exposure to predation. Resting places were confined to a few locations, indicating that turtles may avoid unnecessary energy expenditures when searching for new resting spots. In addition, the turtles may have used the same resting places because they were ideal safe havens from predation pressure. These findings also imply that the turtles were not completely faithful to a single resting place, but used areas that met their needs as a suitable resting place. Consequently, the small number of resting locations may be the result of there being a small number of favourable resting sites. In terms of resting depth, the average was 8.9±3.8 m (range: 3.5–23.8 m) for our turtles. The green turtles nesting at Ascension Island may rest at the maximum depth for neutral buoyancy (MDNB) and in nesting green turtles it was expected to be 17 m [61]. The MDNB of immature turtles would be expected to be less than that of nesting adults, because the lung volume decreases with smaller turtles [62]. Therefore, individuals may select a certain resting depth to make dive durations longer. However, the depths of resting dives in our turtles had a wide range, and especially varied across the resting phases (e.g. CM2 see Fig. 2). Moreover, the depths in several dives were deeper than even the MDNB of nesting turtles. Therefore, our results also indicate that immature green turtles may have a preference for the geographic closeness to seagrass meadows instead of the maximization of dive duration in the selection of a resting place.

According to the video data and behavioral patterns, the main purpose of swimming during other phases would be migration to and from seagrass meadows. A high degree of straightness in the transit between feeding and resting places indicates that the turtles understand the spatial distribution of food and resting resources within the area, and that they minimise the distance travelled along with the costs of locomotion [63]. Another purpose would be to feed on floating invertebrates. Although our video data sometimes showed that turtles seemed to swim aimlessly around their habitats, this might be a prey-searching behavior or the turtles may be assessing their environment for other reasons.

In summary, we demonstrated that immature green turtles maintained high levels of energy by spending as much time feeding as possible and minimising their energy expenditures, spending most of their non-feeding time resting and by selecting resting locations that were close to foraging sites to minimise travel costs. Ultimately, we conclude that these behavioral patterns and time allocations help turtles obtain/conserve energy effectively for growth and consequently maximise their fitness.

## Supporting Information

Table S1 **Recoding periods of the video data logger for each turtle.**
(DOCX)

Table S2 **The result of generalized linear model to investigate the relationship between the occurrence of feeding behavior and time, tidal level or tidal movement.**
(DOCX)

Movie S1 **Feeding behavior on seagrass by an immature green turtle (CM8) in shallow water.**
(WMV)

Movie S2 **Resting behavior of a turtle (CM4) under the reef ledge.** This clip was edited at double speed due to the limitation of file size.
(WMV)

## References

[1] Stearns SC (1992) The Evolution of Life Histories. Oxford: Oxford University Press. 264 p.

[2] Davies NB, Krebs JR, West SA (2012) An introduction to behavioural ecology, 4th Edition. Oxford: Wiley-Blackwell. 520 p.

[3] Martin P, Bateson P (2007) Measuring Behavior: An Introductory Guide, Third Edition. Cambridge: Cambridge University Press. 187 p.

[4] Sakamoto KQ, Sato K, Ishizuka M, Watanuki Y, Takahashi A, et al. (2009) Can ethograms be automatically generated using body acceleration data from free-ranging birds? PLoS ONE, 4, e5379 doi:10.1371/journal.pone.0005379 10.1371/journal.pone.0005379 PMC2671159 19404389

[5] Rutz C, Hays GC (2009) New frontiers in biologging science. Biol lett 5: 289–292. 19324624 10.1098/rsbl.2009.0089 PMC2679933

[6] Fossette S, Schofield G, Lilley MKS, Gleiss AC, Hays GC (2012) Acceleration data reveal the energy management strategy of a marine ectotherm during reproduction. Funct Ecol 26: 324–333 doi: –10.1111/j.1365–2435.2011.01960.x

[7] Houghton JDR, Cedras A, Myers AE, Liebsch N, Metcalfe JD, et al (2008) Measuring the state of consciousness in a free-living diving sea turtle. J Exp Mar Biol Ecol 356: 115–120.

[8] Seminoff JA, Jones TT, Marshall GJ (2006) Underwater behaviour of green turtles monitored with video-time-depth recorders: what’s missing from dive profiles? Mar Ecol Prog Ser 322: 269–280.

[9] MacArthur RH, Pianka ER (1966) On optimal use of a patchy environment. Am Nat 100: 603–609.

[10] Charnov EL (1976) Optimal foraging, the marginal value theorem. Theor Popul Biol 9: 129–136. 1273796 10.1016/0040-5809(76)90040-x

[11] Westoby M (1974) An analysis of diet selection by large generalist herbivores. Am Nat 108: 290–304.

[12] Siegel JM (2005) Clues to the functions of mammalian sleep. Nature 437: 1264–1271. 16251951 10.1038/nature04285 PMC8760626

[13] Newman J (2007) Herbivory. In: Stephens DW, Brown JS, Ydenberg RC, editors. Foraging: behaviour and ecology. Chicago: The university of Chicago press. 175–218.

[14] Navy KA, Shoemaker VH (1984) Field energetics and food consumption of the Galapagos marine iguana, *Amblyrhynchus cristatus* . Physiol Zool 57: 281–290.

[15] Sheppard JK, Marsh H, Jones RE, Lawler IR (2010) Dugong habitat use in relation to seagrass nutrients, tides, and diel cycle. Mar Mamm Sci 26: 855–879.

[16] Valentine JF, Heck KL (1999) Seagrass herbivory: evidence for the continued grazing of marine grasses. Mar Ecol Prog Ser 176: 291–302.

[17] Lobel PS, Ogden JC (1981) Foraging by the herbivorous parrotfish Sparisoma radians. Mar Biol 64: 173–183.

[18] Caceres WC, Fuentes LS, Ojeda FP (1994) Optimal feeding strategy of the temperate herbivorous fish Aplodactylus punctatus: the effects of food availability on digestive and reproductive patterns. Oecologia 99: 118–123. 28313956 10.1007/BF00317091

[19] Bjorndal KA (1980) Nutrition and grazing behavior of the green turtle *Chelonia mydas* . Mar Biol 56: 147–154.

[20] Mortimer JA (1995) Feeding ecology of sea turtles. In: Bjorndal KA, editor. Biology and Conservation of Sea Turtles, Revised edn. Washington DC: Smithsonian Institution Press. 103–109.

[21] Ogden JC, Robinson L, Whitlock K, Daganhardt H, Cebula R (1983) Diel forgaging patterns in juvenile green turtles (*Chelonia mydas* L.) in St. Croix United States Virgin Islands. J Exp Mar Biol Ecol 66: 199–205.

[22] Seminoff JA, Resendiz A, Nichols WJ (2002) Home range of green turtles *Chelonia mydas* at a coastal foraging area in the Gulf of California, Mexico. Mar Ecol Prog Ser 242: 253–265.

[23] Makowski C, Seminoff JA, Salmon M (2006) Home range and habitat use of juvenile Atlantic green turtles (*Chelonia mydas* L.) on shallow reef habitats in Palm Beach, Florida, USA. Mar Biol 148: 1167–1179.

[24] Taquet C, Taquet M, Dempster T, Soria M, Ciccione S, et al (2006) Foraging of the green sea turtle *Chelonia mydas* on seagrass beds at Mayotte Island (Indian Ocean), determined by acoustic transmitters. Mar Ecol Prog Ser 306: 295–302.

[25] MacDonald B, Lewison RL, Madrak SV, Seminoff JA, Eguchi T (2012) Home ranges of East Pacific green turtles, *Chelonia mydas*, in a highly urbanized temperate foraging ground. Mar Ecol Prog Ser 461: 211–221.

[26] Hays GC, Glen F, Broderick AC, Godley BJ, Metcalfe JD (2002) Behavioural plasticity in a large marine herbivore: contrasting patterns of depth utilisation between two green turtle (*Chelonia mydas*) populations. Mari Biol 141: 985–990.

[27] Bjorndal KA, Bolten AB, Chaloupka MY (2003) Survival probability estimates for immature green turtles *Chelonia mydas* in the Bahamas. Mar Ecol Prog Ser 252: 273–281.

[28] Heithaus MR, Frid A, Wirsing AJ, Dill LM, Fourqurean JW, et al (2007) State-dependent risk-taking by green sea turtles mediates top-down effects of tiger shark intimidation in a marine ecosystem. J Anim Ecol 76: 837–844. 17714261 10.1111/j.1365-2656.2007.01260.x

[29] Ministry of the environment government of Japan (2004) Coral reefs in Japan. Tokyo: Japan wildlife research center press. (in Japanese). 375 p.

[30] Biodiversity center of Japan (2008) The report of ecological survey in the neritic waters. Tokyo: Japan wildlife research center press. (in Japanese). 242 p.

[31] Ministry of the environment government of Japan (2006) A survey report of coral population dynamics in Sekisei lagoon. Tokyo: Japan wildlife research center press. (in Japanese). 204 p.

[32] Hamabata T, Nishida S, Kamezaki N, Koike H (2009) Genetic structure of the green turtle (*Chelonia mydas*) in Japan using mtDNA control region sequences. Bull Grad Sch Soc Cul Studies, Kyushu University 15: 35–50.

[33] Nishizawa H, Abe O, Okuyama J, Kobayashi M, Arai N (2011) Population genetic structure and implication for natal philopatry of nesting green turtles *Chelonia mydas* in the Yaeyama Islands, Japan. Endang Species Res 14: 141–148.

[34] Okuyama J, Kawabata Y, Naito Y, Arai N, Kobayashi M (2010) Monitoring beak movements with an acceleration datalogger: a useful technique for assessing the feeding and breathing behaviors of sea turtles. Endang Species Res 10: 39–45.

[35] Watson K, Granger R (1998) Hydrodynamic effect of a satellite transmitter on a juvenile green turtle (*Chelonia mydas*). J Exp Biol 201: 2497–2505. 9698584 10.1242/jeb.201.17.2497

[36] Jones TT, Bostrom B, Carey M, Imlach B, Mickelson J, et al.. (2011) Determining transmitter drag and best-practice attachment procedures for sea turtle biotelemetry studies. NOAA Tech Mem NOAA-NMFS-SWFSC-480.

[37] Tanaka H, Takagi Y, Naito Y (2001) Swimming speeds and buoyancy compensation of migrating adult chum salmon Oncorhynchus keta revealed by speed/depth/acceleration data logger. J Exp Biol 204: 3895–3904. 11807107 10.1242/jeb.204.22.3895

[38] Houghton JDR, Broderick AC, Godley BJ, Metcalfe JD, Hays GC (2002) Diving behaviour during the internesting interval for loggerhead turtles *Caretta caretta* nesting in Cyprus. Mar Ecol Prog Ser 227: 63–70.

[39] Worton BJ (1989) Kernel methods for estimating the utilization distribution in home-range studies. Ecology 70: 164–168.

[40] Seaman DE, Millspaugh JJ, Kernohan BJ, Brundige GC, Raedeke KJ, et al (1999) Effects of sample size on kernel home range estimates. J Wildl Manag 63: 739–747.

[41] Batschelet E (1981) Circular statistics in biology, Academic Press, New York.

[42] R Development Core Team (2012) R: A Language and Environment for Statistical Computing. Vienna: R Foundation for Statistical Computing.

[43] Bjorndal KA, Bolten AB, Chaloupka MY (2000) Green turtle somatic growth model: evidence for density dependence. Ecol Appl 10: 269–282.

[44] Yamamoto T, Touma T (1995) Stomach contents survey on sea turtle *Chelonia mydas* and *Eretmochelys imbricata* . Ann Rep Okinawa Pref Fish Exp Station 57: 227–236.

[45] Burkholder D, Heithaus MR, Thomson JA, Fourqurean J (2011) Diversity in trophic interactions of green sea turtles *Chelonia mydas* on a relatively pristine coastal foraging ground. Mar Ecol Prog Ser 439: 277–293.

[46] Lemons G, Lewison RL, Komoroske L, Gaos AR, Lai CT, et al (2011) Trophic ecology of green sea turtles in a highly urbanized bay: Insights from stable isotopes and mixing models. J Exp Mar Biol Ecol 405: 25–32.

[47] Heithaus MR, McLash JJ, Frid A, Dill LM, Marshall GJ (2002) Novel insights into green sea turtle behaviour using animal-borne video cameras. J Mar Biol Assoc UK 82: 1049–1050.

[48] MacDonald BD, Lewison RL, Madrak SV, Seminoff JA, Eguchi T (2013) Seasonal and diel variability in site visitation and movement behavior of East Pacific green turtles, *Chelonia mydas*, in a highly urbanized temperate foraging ground. J Exp Mar Biol Ecol 443: 56–64.

[49] Mendonca MT (1983) Movements and feeding ecology of immature green turtles (*Chelonia mydas*) in a Florida lagoon. Copeia 1983: 1013–1023.

[50] Williams SL (1988) Thalassia testudinum productivity and grazing by green turtles in a highly disturbed seagrass bed. Mar Biol 98: 447–455.

[51] Bailey DW, Gross JE, Laca EA, Rittenhouse LR, Coughenour MB, et al (1996) Mechanisms that result in large herbivore grazing distribution patterns. J Range Manage 49: 386–400.

[52] Chilvers BL, Delean S, Gales NJ, Holley DK, Lawler IR, et al (2004) Diving behaviour of dugongs, *Dugong dugon* . J Exp Mar Biol Ecol 304: 203–224.

[53] van Gils JA, Schenk IW, Bos O, Piersma T (2003) Incompletely informed shorebirds that face a digestive constraint maximize net energy gain when exploiting patches. Am Nat 161: 777–793. 12858284 10.1086/374205

[54] Bjorndal KA (1985) Nutritional ecology of sea turtles. Copeia 1985: 736–751.

[55] Swimmer Y, Arauz R, Higgins B, McNaughton L, McCracken M, et al (2005) Food color and marine turtle feeding behavior: Can blue bait reduce turtle bycatch in commercial fisheries? Mar Ecol Prog Ser 295: 273–278.

[56] Balazs GH, Forsyth RG, Kam AKH (1987) Preliminary assessment of habitat utilization by Hawaiian green turtles in their resident foraging pastures. NOAA Tech Mem NMFS-SWFC-71. US Department of Commerce, Washington, DC.

[57] Godley BJ, Lima EHSM, Akesson S, Broderick AC, Glen F, et al (2003) Movement patterns of green turtles in Brazilian coastal waters described by satellite tracking and flipper tagging. Mar Ecol Prog Ser 253: 279–288.

[58] Schofield G, Hobson VJ, Lilley MKS, Katselidis KA, Bishop CM, et al (2010) Inter-annual variability in the home range of breeding turtles: Implications for current and future conservation management. Biol Cons 143: 722–730 doi:10.1016/j.biocon.2009.12.011

[59] Bradshaw CJA, Sims DW, Hays GC (2007) Measurement error causes scale-dependent threshold erosion of biological signals in animal movement data. Ecol Appl 17: 628–638. 17489266 10.1890/06-0964

[60] Brown JS (1988) Patch use as an indicator of habitat preference, predation risk and competition. Behav Ecol Sociobiol 22: 37–47.

[61] Hays GC, Metcalfe JD, Walne AW (2004) The implications of lungregulated buoyancy control for dive depth and duration. Ecology 85: 1137–1145.

[62] Hochscheid S, McMahon CR, Bradshaw CJA, Maffucci F, Bentivegna F, et al (2007) Allometric scaling of lung volume and its consequences for marine turtle diving performance. Comp Biochem Physiol. 148: 360–367. 10.1016/j.cbpa.2007.05.010 17596981

[63] Ford RG (1983) Home range in a patchy environment: optimal foraging predictions. Am Zool 23: 315–326.

