# Correction: Ethogram of Immature Green Turtles: Behavioral Strategies for Somatic Growth in Large Marine Herbivores

**DOI:** 10.1371/annotation/2654a180-d8b2-4516-baa5-be4806676c62

**Published:** 2013-07-03

**Authors:** Junichi Okuyama, Kana Nakajima, Takuji Noda, Satoko Kimura, Hiroko Kamihata, Masato Kobayashi, Nobuaki Arai, Shiro Kagawa, Yuuki Kawabata, Hideaki Yamada

Figures 1 and 2 were incorrectly switched. The figure appearing as Figure 1 belongs with the title and legend of Figure 2, and the figure appearing as Figure 2 belongs with the title and legend of Figure 1. The titles and legends themselves are in correct order. 

