# Supplementary material for: Ethogram of Immature Green Turtles: Behavioral Strategies for Somatic Growth in Large Marine Herbivores
Source: PLoS One. 2013 Jun 19;8(6):e65783. doi: 10.1371/journal.pone.0065783 (PMC3686772; doi:10.1371/journal.pone.0065783)
Supplement: Table S1 — Recoding periods of the video data logger for each turtle. (DOCX) [file pone.0065783.s001.docx]

**Table S1. Table S1. Recoding periods of the video data logger in each turtle.**

| **Turtle** | **CM 3** | **CM 4** | **CM 5** | **CM 6** | **CM 8** |
| --- | --- | --- | --- | --- | --- |
| 1 | 7/7 5:30-9:30 | 7/7 11:30-12:00 | 8/3 19:00–5:30 | 8/17 14:00-14:40 | 8/28 16:00-17:25 |
| 2 |  | 7/7 13:00-13:30 |  |  | 8/29 08:30-12:01 |
| 3 |  | 7/7 14:30-15:00 |  |  |  |
| 4 |  | 7/7 16:00-16:30 |  |  |  |
| 5 |  | 7/7 17:30-18:00 |  |  |  |
| 6 |  | 7/8 11:30-12:00 |  |  |  |
| 7 |  | 7/8 13:00-13:30 |  |  |  |
| 8 |  | 7/8 14:30-15:00 |  |  |  |
| 9 |  | 7/8 16:00-16:30 |  |  |  |
| 10 |  | 7/8 17:30-18:00 |  |  |  |
