# Supplementary material for: Ethogram of Immature Green Turtles: Behavioral Strategies for Somatic Growth in Large Marine Herbivores
Source: PLoS One. 2013 Jun 19;8(6):e65783. doi: 10.1371/journal.pone.0065783 (PMC3686772; doi:10.1371/journal.pone.0065783)
Supplement: Table S2 — The result of generalized linear model to investigate the relationship between the occurrence of feeding behavior and time, tidal level or tidal movement. (DOCX) [file pone.0065783.s002.docx]

**Table S2. The result of generalized linear model to investigate the relationship between the occurrence of feeding behavior and time, tidal level or tidal movement.**

|  | **Estimate** | **S.E.** | ***z* value** | ***P*** |
| --- | --- | --- | --- | --- |
| Intercept | -2.39 | 1.56 | -1.53 | 0.13 |
| 0-1 h | 0.20 | 1.46 | 0.14 | 0.89 |
| 1-2 h | 0.28 | 1.47 | 0.19 | 0.85 |
| 2-3 h | 1.09 | 1.29 | 0.85 | 0.40 |
| 3-4 h | 1.01 | 1.29 | 0.79 | 0.43 |
| 4-5 h | 1.57 | 1.23 | 1.28 | 0.20 |
| **5-6 h** | **3.22** | **1.14** | **2.81** | **0.00** |
| **6-7 h** | **5.05** | **1.23** | **4.12** | **0.00** |
| **7-8 h** | **4.62** | **1.19** | **3.89** | **0.00** |
| **8-9 h** | **3.40** | **1.14** | **2.99** | **0.00** |
| 9-10 h | 2.05 | 1.17 | 1.75 | 0.08 |
| 10-11 h | 1.42 | 1.22 | 1.17 | 0.24 |
| 11-12 h | 1.50 | 1.22 | 1.23 | 0.22 |
| 12-13 h | 0.44 | 1.47 | 0.30 | 0.76 |
| 13-14 h | 0.55 | 1.48 | 0.37 | 0.71 |
| 14-15 h | -14.08 | 904.33 | -0.02 | 0.99 |
| 15-16 h | 1.32 | 1.31 | 1.01 | 0.31 |
| 16-17 h | 2.26 | 1.22 | 1.86 | 0.06 |
| **17-18 h** | **3.65** | **1.17** | **3.12** | **0.00** |
| **18-19 h** | **4.09** | **1.17** | **3.49** | **0.00** |
| **19-20 h** | **3.08** | **1.15** | **2.68** | **0.01** |
| 20-21 h | 1.31 | 1.22 | 1.08 | 0.28 |
| 21-22 h | 0.80 | 1.28 | 0.63 | 0.53 |
| 22-23 h | 0.70 | 1.27 | 0.55 | 0.59 |
| Tidal level | 0.01 | 0.00 | 1.42 | 0.16 |
| Rising tide | -1.59 | 1.26 | -1.26 | 0.21 |
| Falling tide | -1.48 | 1.26 | -1.18 | 0.24 |

Estimate represents the coeficient estimate of each vaiable in regression fuction; S.E., standard error.
